# Supervised exercise training versus usual care in ambulatory patients with left ventricular assist devices: A systematic review

**DOI:** 10.1371/journal.pone.0174323

**Published:** 2017-03-31

**Authors:** Harsha V. Ganga, Amanda Leung, Jennifer Jantz, Gaurav Choudhary, Loren Stabile, Daniel J. Levine, Satish C. Sharma, Wen-Chih Wu

**Affiliations:** 1 Division of Cardiology, Medical Service, Veterans Affairs Medical Center, Providence, Rhode Island, United States of America; 2 Division of Cardiology, Dept. of Medicine, The Warren Alpert Medical School of Brown University, Providence, Rhode Island, United States of America; 3 Center for Cardiac Fitness, Miriam Hospital, Providence, Rhode Island, United States of America; Ospedale del Cuore G Pasquinucci Fondazione Toscana Gabriele Monasterio di Massa, ITALY

## Abstract

Implantation of left ventricular assist devices (LVAD) has increased because of improved safety profile and limited availability of heart transplantation. Although supervised exercise training (ET) programs are known to improve exercise capacity and quality of life (QoL) in heart failure (HF) patients, similar data is inconclusive in LVAD patients. Thus, we performed a systematic review on studies that incorporated supervised ET and measured peak oxygen uptake in LVAD patients. A total of 150 patients in exercise and 55 patients in control groups were included from 8 studies selected from our predefined criteria. Our systematic review suggests supervised ET has an inconsistent effect on exercise capacity and QoL when compared to control groups undergoing usual care. A quantitative sub-analysis was performed with 4 studies that provided enough data to compare peak oxygen uptake and QoL at baseline and at follow-up. After at least 6 weeks of training, LVAD patients undergoing supervised ET demonstrated significant improvement in exercise capacity (standardized mean difference [SMD] = 0.735, 95% Confidence Interval-[CI], 0.31–1.15 units of the standard deviation, P = 0.001) and QoL scores (SMD = 1.58, 95% CI 0.97–2.20 units of the standard deviation, P <0.001) when compared to the usual care group, with no serious adverse events with exercise. These results suggest that supervised ET is safe and can improve patient outcomes in LVAD patients when compared to the usual care.

## Introduction

Exercise intolerance is a cardinal feature of advanced heart failure (HF) patients. Exercise training (ET) was successfully shown to improve exercise capacity, quality of life (QoL) and HF symptoms in patients with HF.[1, 2] For patients with end-stage HF, implantation of left ventricular assist device (LVAD) has steadily increased due to their improved safety and the limited donor availability for heart transplantation.[3] Indeed, LVADs are being used as destination therapy (DT) in an increasing number of HF patients.[4] Despite LVAD implantation, HF symptoms are often not relieved, with some patients continuing to have poor exercise capacity due to prolonged immobility and skeletal myopathy.[5], [6] In addition, LVAD patients may not always experience improved QoL due to poor muscle strength,[7] and cannot perform simple, short-duration daily activities that require less than 2 minutes of muscle activation like showering or dressing.[7] Furthermore, decreased muscle strength is an independent predictor of mortality in HF.[8]

Lack of guidance and absence of uniform supervision are important barriers to initiation and continuation of exercise therapy in cardiac patients.[9] Medically supervised exercise programs have a structured regimen and are monitored by professionals. The difference between supervised and unsupervised ET is related to better patient adherence and greater intensity of exercise.[10]

Despite data showing ET enhances skeletal muscle strength and improves functional capacity and QoL in HF patients, [7, 11] the data on efficacy of supervised ET in LVAD patients is limited by small sample size with conflicting results. The role of supervised ET in improving exercise capacity and QoL in LVAD patients as compared to usual care remains controversial. Therefore, we conducted this systematic review with the primary objective of evaluating the effect of supervised ET on exercise capacity and QoL in LVAD patients.

## Methods

### Data sources

**Fig 1:**
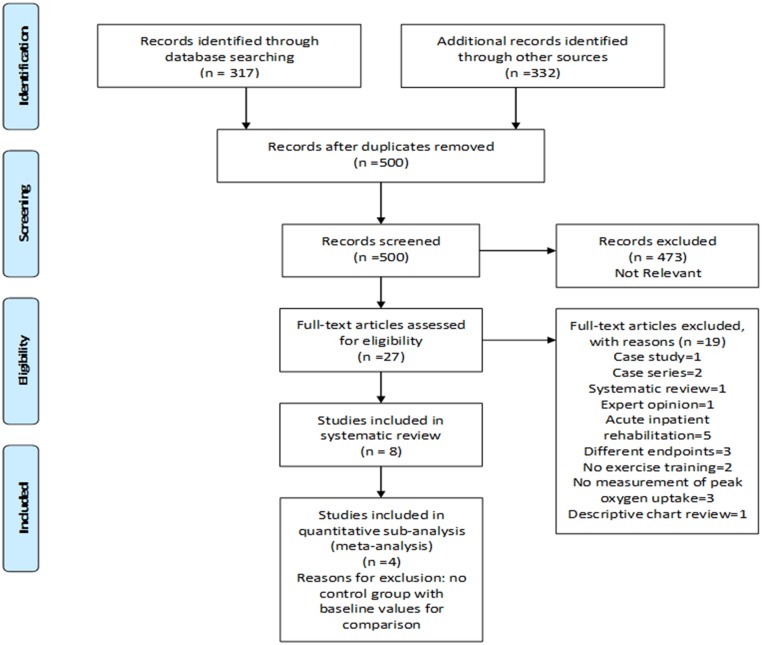
Flow diagram depicting study selection.

We performed electronic searches in 7 databases from their inception to December 2015: PubMed, Embase, EBSCO Rehabilitation and Sports Medicine Source, Web of Science, Cochrane Central Registry of Controlled Trials, Cochrane Database of Systematic Reviews, Google Scholar according to PRISMA guidelines (Fig 1 and S1 File). We used the following MeSH terms and keywords: heart-assist devices, ventricular-assist devices, exercise, cardiovascular rehabilitation, and exercise training to identify all the available trials with maximum sensitivity. We used a librarian’s expertise (CB) for searching unpublished reports and thesis work. Only English language papers were included.

### Study selection and data extraction

Due to the scarcity of data on our chosen topic, studies were required to have supervised ET alone or in combination with standard medical care or in the setting of cardiac rehabilitation. For accurate assessment of ET on exercise capacity, only studies that measured peak oxygen uptake (ml/kg/min) were included. HG and AL independently reviewed all studies from the search list and independently reported results in separate data sheets. When disparities arose regarding study selection and inclusion, consensus was achieved with the assistance of a third independent investigator (WW). Study investigators [12, 13] were contacted through email to retrieve missing data when appropriate. We considered both RCTs and observational studies. Data was extracted for baseline characteristics that included age, gender, follow-up period, ejection fraction, patients with ischemic (ICM) versus nonischemic cardiomyopathy(NICM), type of LVADs, time to ET intervention after LVAD implantation, and baseline values of peak oxygen uptake and QoL parameters. We collected data on sample size, means, and standard deviations (at baseline and follow-up) for the outcome measurements. We calculated dose of aerobic ET as dose = number of weeks of exercise x average sessions per week x duration of each session in minutes.[14] Although pulsatile-flow LVADs are in minimal use currently, we included them in this review to examine differences in outcome (versus continuous-flow devices) and whether having pulsatility provided any advantage with respect to either improvement in exercise capacity or adverse events.

### Quality assessment

Two independent reviewers HG and AL evaluated the quality of the selected studies, on separate pre-specified forms. We used Downs and Black instrument[15] to assess quality of individual studies for sub-analysis. Downs and Black instrument uses 27 criteria to assess quality of reporting, internal validity (bias and confounding), and external validity (generalizability) and is listed in the top six quality assessment scales for systematic reviews.[16]

### Statistical analysis

For the sub-analysis performed in this systematic review, we used a random effects model (DerSimonian and Laird method) to account for clinical diversity and methodological variation among the pooled studies. We computed standardized mean differences (SMD) and corresponding 95% CI for all continuous outcomes to standardize the results of individual studies to a uniform scale before they were combined. SMD is used as a summary statistic when all the studies measure the same outcome but use different tools (or scales) and is calculated as the ratio of the difference in the mean outcome between groups and the standard deviation of the outcome among participants.[17] Results are expressed as a proportion or units of the standard deviation. We conducted sensitivity analysis by excluding one study from the pool each time for primary outcome (peak oxygen uptake) to determine the robustness of our results. We assessed for heterogeneity of SMD across studies by using *I*^*2*^ test which is equal to the percentage of total variation across all studies as a result of heterogeneity reported for each analysis, so as to render inter-study heterogeneity interpretable. *I*^*2*^ more than 50% was considered substantial heterogeneity. Meta-regression was done to evaluate if baseline differences in peak oxygen uptake, the setting where supervised ET took place, the duration of supervised ET, age, strength training inclusion or time of ET initiation after LVAD implant influenced the pooled SMD. Publication bias was evaluated using a funnel plot and quantified using Egger test. All P values were two-tailed and considered significant at α <0.05. Confidence intervals were reported at 95%. We used Comprehensive Meta-Analysis Software (CMA software, version 3.2, Biostat Inc, Englewood, NJ, 2014) for conducting this meta-analysis.

## Results

Our search resulted in 3 RCTs[12, 18, 19] and 2 prospective observational studies[13, 20] and 3 retrospective observational studies.[21–23] We obtained full-text articles for the included studies. We summarized baseline demographic and clinical characteristics in Table 1. Most studies were relatively small in sample size (median 19, 11–70). One study[22] had no control group. Three studies compared ET in LVAD patients with either HF patients[23] or artificial heart or heart transplant patients.[20, 21] In total, there were 150 patients in the ET group and 55 in the control group. The age of the patients varied from 37 to 63 years. Although 6 of the 8 studies included women, they accounted for only 16.66% of the patients recruited overall (Table 1). However, the proportion of women in the most recent studies[22, 23] improved ranging from 24 to 37%. Four studies reported ejection fraction which varied by 13 to 21%.[12, 18, 20, 23]

**Table 1:** Description of the ET group characteristics from the included studies.

Study/Year	Design	N	Mean age (Y)	M/F	EF	ICM/NICM	BT/ DT	PF/CF	Time to ET	ETduration(W)	SPW/DES	ETDose‡(Mi)	ETS	AE
**De Jonge,2001**	P	15	37(±12)	15/0	13±5	7 /8	15/ 0	15/0	2 weeks	12	3-5/20-40	720–2400	HP	None
**Laoutaris,2011**	RCT	10	37.2±17.7	10/0	NA	0/10	10/0	13/2	6.6 (±4.4)Mn	10	3-5/30-45	900–2200	HM+HP	None
**Hayes,2012**	RCT	7	48.7±14.5	6/1	16±5	4/3	7/ 0	0/ 7	NA	8	3/30	1440	HP	None
**Kugler,2012**	P	34	52.2±2	29/5	NA	15/19	34/0	0/34	6 weeks	18±	4/20	720	HM	None
**Karapolat,2013**	R	11	46±14	10/1	NA	NA /6	11/ 0	3/ 11	NA	8	3/30	720	HP	None
**Compostella,2014**	R	26	63.4±7.4	23/3	20±6	NA	0/26	0/26	34±20 days	2	6/NA	-	HP	None
**Kerrigan,2014**	RCT	16	53±13	11/7	21±7	6/12	NA	0/ 26	82 days(avg)	6	3/30	540	HP	Syncope
**Marko,2014**	R	41	55 (±12)	33/8	NA	19/17	NA	0/ 41	48 (±38)days	4.6	NA	-	HP	NSVT

AE = adverse events, Avg = average, BT = bridge to transplant, CF = continuous-flow, DT = destination therapy, ET = exercise training, ETS = exercise training setting, F = female, P = prospective, PF = pulsatile-flow, R = retrospective, RCT = randomized control trial, M = male, Mi = minutes, Mn = months, NA = not available, ICM = ischemic cardiomyopathy, NICM = nonischemic cardiomyopathy, NA = not available, NSVT = nonsustained ventricular tachycardia, SPW/DES = sessions per week and duration of each session in minutes, W = weeks, ± = lowest possible time period close to the included studies extrapolated from the graph,

^‡^ = aerobic ET dose calculating by multiplying number of weeks of training x session /week x duration of each session (units = minutes)

Six studies reported diagnosis leading to LVAD implantation (Table 1). Overall LVAD was implanted in 51 patients with ICM and 75 patients with NICM. None of the studies compared ICM patients with NICM patients for either outcomes or adverse events. Six studies whether LVAD placement are for destination therapy or bridge-to-transplant therapy (Table 1). Of those 6 studies, 5[13, 18–21] included only patients with LVADs implanted as a bridge-to-transplant. None of the studies compared ET in LVADs used as bridge-to-transplant versus destination therapy.

### Quality assessment results

We assessed quality of the pooled studies using Downs and Black criteria (Table 2).[15] The mean Down and Black quality assessment score was 21.4 (±3) with 7 studies [12, 18–21, 23, 24] having good to excellent quality and 1 study[13] having fair quality.

**Table 2:** Downs and Black quality assessment score.

Downs and Black Criteria	De Jonge et al.	Laoutaris et al.	Hayes et al.	Kugler et al.	Karapolat et al.	Compostella et al.	Kerrigan et al.	Marko et al.
Reporting	9	9	10	8	9	9	10	10
External Validity	3	2	3	2	3	3	3	3
Bias	4	6	6	4	4	4	6	4
Confounding	4	5	6	2	4	4	6	4
Power	0	0	1	1	0	0	0	0
Total Score	20	22	26	17	20	20	25	21

Quality levels of Downs and Black scores: excellent (26 to 28), good (20 to 25), fair (15 to 19), and poor (≤14)

### Types of LVAD

All the studies reported the type of LVAD implanted (continuous-flow versus pulsatile-flow, Table 1). Five studies included only continuous-flow devices.[12, 13, 18, 22, 23] One study[20] included only pulsatile-flow devices and two[19, 21] included both pulsatile and continuous-flow devices. In total, pulsatile-flow devices were implanted in 31 patients and continuous-flow devices were implanted in 147 patients. None of the studies compared ET in continuous-flow devices versus pulsatile-flow devices for exercise capacity, QoL, or adverse events.

### Inter-agency Registry for Mechanically Assisted Circulatory Support (INTERMACS) levels

Five studies reported INTERMACS levels.[12, 18, 19, 22, 23] In 3 studies[12, 18, 19] with a control LVAD group, there was no significant difference in the INTERMACS levels between the treatment and control groups. In one study[23] that compared LVAD group with advanced HF group, there was a significant difference in the INTERMACS levels between the LVAD group and the HF group [2.5(±0.8) versus 4.2 (±0.5) P<0.001].

### Duration of ET

The duration of ET is reported in Table 1. The duration of ET varied from 2 weeks[23] to 18 weeks.[13] In a study by Kugler and coworkers,[13] LVAD patients underwent ET for the longest duration with a significant improvement in peak oxygen uptake in the treatment group as compared to the control group. In 3 other studies,[12, 18, 19] that had duration of ET between 6 to 12 weeks, there was no significant improvement in the peak oxygen uptake between treatment group and control group at the end of the follow-up.

### Location and supervision of ET

The location of ET is reported in Table 1. The supervision of ET included direct observation in the cardiac rehabilitation gym setting[12, 18] or monitoring of exercise intensity and frequency through a home monitoring system [use of smart card to store mean training heart rate, mean training workload and rate of perceived exertion][13] or both (implementation of supervised ET protocol at home combined with hospital visits (2–3 times/week) for direct supervision of inspiratory muscle training).[19] Only 2 studies[12, 22] reported telemetry monitoring of heart rhythm. None of the studies compared differences in exercise capacity or QoL between ET at home-based versus hospital-based programs.

### Interventions and dose of ET

Interventions employed in ET are described in Table 3. The primary mode of intervention was mostly aerobic ET. Three studies incorporated only aerobic ET.[12, 13, 23] Four studies incorporated strength training exercises, in addition to aerobic ET.[18, 20–22] One study[19] performed inspiratory muscle training. The dose of ET varied considerably across studies, in total duration (2 to 18 weeks), frequency (3 to 6 sessions/week), session length (20 to 90 minutes/session), and intensity (Bjorg RPE 12–14; 60 to 80% of heart rate reserve; 60 to70% peak oxygen uptake, Table 3) We calculated dose of aerobic ET when possible and it ranged from 540 to 2400 minutes (Table 1). None of the studies reported problems with adherence to ET and no study reported higher adherence with hospital-based program as compared to a home-based program. Recommendations on diet and/or exercise per usual standards were given for the control groups present in 4 studies (Table 3); however, there was no structured monitoring or supervision to ensure accomplishment of the tasks in the control group. We are unable to quantify the precise level of intervention in the control group due to inconsistent reporting and lack of restriction on exercise limits in the control group.

**Table 3:** Exercise intervention used in supervised ET and control groups.

Study	Supervised ET Group	Control Group
**De Jonge,**[20]**,2001**	2 to 6 min of low level activities alternated with 1 to 2 min of rest. Training sessions with bicycle, treadmill, and rowing machine. Intensity increases based on Bjorg RPE. Duration of exercise gradually increased to 20–40 min/day 3–5 times a week. Strength and endurance training of local muscle groups.	-
**Laoutaris,**[19]**,2011**	Walk every day for 30–45 min on their own.Participants exercised at home, using bike or treadmill, for 30–45 min at moderate intensity level of 12–14 on Bjorg RPE, 3 to 5 days a week. In addition, they underwent high-intensity inspiratory muscle training (IMT) 2 to 3 times week in the hospital. Exercise sessions were quantified by confirmation of implementation of home ET protocol during each IMT session 2 to 3 times a week	Walk every day for 30–45 minutes on their own
**Kugler,**[13]**,2011**	Home-based, tailored, every other day, smartcard-guided, cycle ergometer training program supplemented by regular phone calls for psychosocial support and training updates. Exercise sessions were quantified by recording training data (mean training heart rate, mean training workload and RPE) in a smartcard and is based on a protocol in a study by Tegtbur et al.[25]	Recommendations to be on healthy diet, maintain normal range BMI, improve physical fitness by exercising regularly, and psychosocial support as needed.
**Hayes,**[18]**,2012**	Participated in Mobilization Protocol (see control group) on days when they did not attend gym. Physiotherapy in gym for 1 hour, 3 days a week for 8 weeks; initially as inpatients, and continuing after hospital discharge. Exercise training included 15 minutes on treadmill, 15 min on stationary bike, and 3 Upper Extremity and Lower Extremity strength training exercises aiming for 2 sets of 10 repetitions. Workload intensity progressed based RPE and dyspnea.	Mobilization Protocol: Participants instructed to progressively increase the distance they walked each day, on their own, maintaining moderate intensity Bjorg RPE of 13. Participants to walk a minimum of 5 days. Overall aim is to increase walk to 60 minutes.
**Karapolat,**[21]**,2013**	Flexibility exercises (range of motion, stretching exercise), aerobic sessions lasting 30 minutes, 60–70% of peak VO2, and 12–14 Bjorg RPE, strengthening exercises involving UE and LE muscle groups, breathing exercises and relaxation exercises. Exercise sessions for 90 min, occurring 3 times a week for 8 weeks	-
**Compostella,**[23]**,2014**	Three daily sessions of exercise-based training for 6 days a week. Exercise training includes breathing exercises, aerobic training, and calisthenics.	-
**Kerrigan,**[12]**,2014**	Supervised exercise training program 3 days a week for 6 weeks, completed primarily by treadmill and a secondary modality (cycle ergometer, recumbent stepper) for 30 minutes at a training intensity set at 60% of the heart rate reserve, with patients allowed to progress to an intensity of 80% heart rate reserve	Daily walking with follow-up calls at weeks 2, 4 and 6 on their own
**Marko,**[22]**,2014**	Aerobic training with bicycle ergometer and included interval training consisting of alternating high and low periods of training and 3 min warm up and cool down periods. Strength training directed on LE muscles only, with 2 series of 12 repetitions each. Walking training and gymnastics training with coordination, strength and balance training exercises.	-

ET: exercise training group, RPE: rate perceived exertion, VAD: ventricular assist device, UE: upper extremity, LE: lower extremity

### Supervised ET effect on peak oxygen uptake

Six studies reported peak oxygen uptake in ml/kg/min at baseline and at follow-up.[12, 13, 18, 19, 21, 22] In 5 of those 6 studies, there is consistent evidence for significantly improved peak oxygen uptake in the treatment group from baseline to follow-up.[12, 18, 19, 21, 22] Four studies had control group with LVADs undergoing usual care without supervised ET and we summarized baseline and clinical characteristics of these 4 studies in Table 4. These 4 studies reported peak oxygen uptake at baseline and follow-up. Data was extrapolated from the graph in one study.[13] When compared with the control group at the end of the follow-up in those 4 studies, ET significantly improved peak oxygen uptake in only 1 study.[13] The level of the peak oxygen uptake achieved varied from 12.5 ml/kg/min to 24.2 ml/min/kg. Peak oxygen uptake was calculated using cardiopulmonary exercise testing on a treadmill in 4 studies [12, 19–21] and on a bicycle ergometer in 4 studies.[13, 18, 22, 23] In another study,[20] peak oxygen uptake was measured when patients already underwent ET for few weeks prior to checking baseline peak oxygen uptake. One study[23] investigated whether peak oxygen uptake was related to ejection fraction and did not find any correlation.

**Table 4:** Baseline characteristics of studies included in the quantitative sub-analysis.

Criteria	Laoutaris et al[19]			Kugler et al[13]			Hayes et al[18]			Kerrigan et al[12]		
Study Design	RCT			Prospective NRS			RCT			RCT		
Follow-up Period	10 weeks			18 weeks*			8 weeks			6 weeks		
	ET	Ctrl	P-value	ET	Ctrl	P-value	ET	Ctrl	P-value	ET	Ctrl	P-value
Patients	10	5	-	34	36	-	7	7	-	16	7	-
Mean Age (yrs.)	37.2(±17.7)	41.8(±14.6)	0.90	52.2(±2)	51 (±2)	0.16	48.7(±14.5)	45.9 (±14.6)	0.72	53 (±13)	60 (±12)	0.23
Gender			0.20			0.13			1.00			0.30
Male	10	4		29	32		6	6		9	7	
Female	0	1		5	4		1	1		7	1	
BMI (kg/m^2^)	24.5 (±3.3)	23.2 (±5.5)	0.60	24 (±0.60)	24(±0.60)	0.34	NA	NA	-	27(±5)	27(±4)	0.80
EF (%)	NA	NA		NA	NA		16(±5)	13.3(±4.4)	0.31	21(±7)	21(±9)	0.87
Diagnosis leading to LVAD			1.00			0.58			0.21			0.67
ICM	0	0		15	18		4	1		6	2	
NICM	10	5		19	18¶		3	6		12	6	
INTERMACs			0.50						0.59			0.48
I	1	2		NA	NA		-	4		4	0	
II	7	2		NA	NA		4	3		2	1	
III	2	1		NA	NA		-	-		8	6	
IV	-	-		NA	NA		-	-		4	1	
V	-	-		NA	NA		-	-		-	-	
ET initiation after LVAD Implant	6.6 (±4.4) M	5.6(±3.8)M	0.60	6 weeks			NA			1–6 months		
Baseline Peak Vo2 (ml/kg/min)	16.8(±3.7)	14.9(±4)	0.50	18.5(±0.8)	16.3(±0.6)	0.17	10.5(±2.3)	12.4(±1.7)	0.10	13.6(±3.3)	11.2(±2.2)	NA
Baseline 6-min walk (meters)	462(±88)	430(±76)	NA	NA	NA	NA	351(±77)	361(±129)	0.77	350(±64.7)	336(±59)	NA
Modality for exercise	Bike or TM			Cycle ergometer			Cycle ergometer			TM (primary),SC,AE,RS		
LVADs used in the study	Pulsatile and continuous-flow LVADs†			Continuous-flow LVADs			Continuous-flow LVADs			Continuous-flow LVADS		
Scale used to assess QoL	Minnesota Living with HF Questionnaire‡			Short-Form Health Survey (SF-36)			SF-36			KCCQ		
Exercise Setting	Home-based and hospital facility			Hospital facility (gym)			Home-based			Hospital facility		

AE = arm ergometer, BMI = Body Mass Index, Ctrl = Control group, ET = exercise training, group, HF = heart failure, K = Kansas city cardiomyopathy questionnaire, M = months, NA = not available, NRS = non-randomized study, RCT = randomized controlled trial, Rx* = total 18 months, from baseline evaluation at 6 weeks, measurements done at 18 weeks from baseline evaluation were used for comparison with other studies in the meta-analysis, RS = recumbent stepper, SC = stationary cycle, TM = treadmill,

^†^ = 13/15 devices are pulsatile volume displacement devices,

^‡^ = reverse coded to match the scale of other questionnaires,

^¶^ = one patient with myocarditis.

Only 4 studies [13, 18, 22, 23] reported whether pump speed of continuous-flow devices was adjusted during exercise or for exercise testing to measure peak oxygen uptake. In 2 studies[18, 23], LVAD pump speed was not adjusted. In 1 study,[13] LVAD pump speed was adjusted individually during exercise testing in order to allow maximum support. In another study,[22] small speed changes were performed in 3 patients with HeartWare devices during their stay in the rehabilitation center.

**Fig 2:**
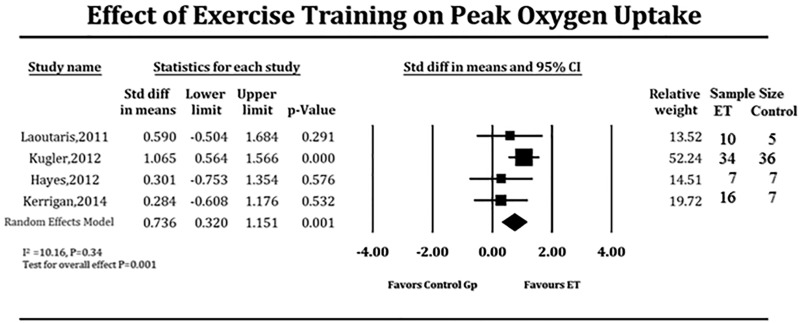
Forest plot depicting effect of supervised exercise training on exercise capacity measured as peak oxygen uptake (ml/kg / min). ET = exercise training, Gp = group.

**Fig 3:**
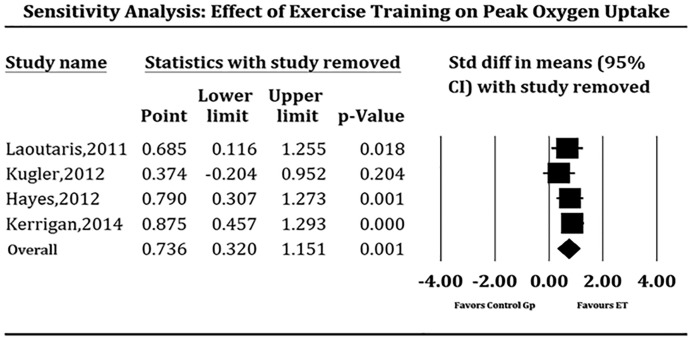
Forest plot depicting sensitivity analysis of studies pooled to analyze effect of supervised exercise training on exercise capacity measured as peak oxygen uptake (ml/kg per min). ET = exercise training, Gp = group.

Due to the strong likelihood of the results being influenced by small sample size, we pooled studies that specifically reported peak oxygen uptake in ml/kg/minute at baseline and at follow-up for comparison with a control LVAD group which did not undergo supervised ET. This quantitative sub-analysis could be performed in only 4 studies (Table 4). After 6 to 18 weeks of supervised ET, LVAD patients demonstrated modest but significant improvement in exercise capacity when compared to those who did not (SMD 0.736, 95% CI 0.32–1.15 units of the standard deviation, P = 0.001, Fig 2), which translated to 1.46 ml/kg/min improvement of the supervised ET over the control group. There is low heterogeneity among the pooled studies (*I*^*2*^ = 11.48, P = 0.33). Meta-regression demonstrated no significant effect on baseline peak oxygen uptake values (coefficient = 0.73, P = 0.39), time of ET initiation after LVAD implant (coefficient = 0.12, P = 73), location of supervised ET (coefficient = 2.74, P = 0.09), duration of supervised ET (coefficient = 3.29, P = 0.07), inclusion of strength training in the regimen (coefficient = 0.66, P = 0.42), dose of aerobic ET (coefficient = 0.20, P = 0.66), and age (coefficient = 0.05, P = 0.83) on the results of the pooled SMD. We conducted sensitivity analysis to examine if effects of supervised ET on peak oxygen uptake differed as a function of study design or type of exercise intervention by eliminating each study from four-study pool, one study at a time (Fig 3); results of which showed consistent benefits of supervised ET on exercise capacity (peak oxygen uptake) in LVAD patients. No significant publication bias was observed in funnel plot and the Egger’s test (P = 0.10).

### Supervised ET effect on submaximal exercise capacity

Three studies [12, 18, 19] assessed the submaximal exercise capacity in LVAD patients with a 6-minute walk test. ET significantly improved 6-minute walk test from baseline to follow-up within the treatment group in all 3 studies.[12, 18, 19] However, there was no difference in the 6-minute walk test distance when compared to the control group at the end of follow-up.[12, 18, 19]

### Supervised ET effect on muscle strength

Four studies incorporated strength training exercises but did not measure change in muscle strength at the end of the follow-up.[18, 20, 21, 23] One study[12] measured change in muscle strength but did not include strength training exercises. This study[12] demonstrated 17% improvement in peak leg torque as compared to no change in the control group without supervised ET.

### Supervised ET effect on QoL

Five studies reported the effect of supervised ET on QoL at baseline and follow-up (Table 4). Two studies [13, 18] used the Short Form-36 scale to assess QoL. One study [19] used the Minnesota Living with Heart Failure Questionnaire and another used the Kansas City Cardiomyopathy Questionnaire to assess QoL. In 4 studies,[12, 13, 18, 19] LVAD patients who underwent supervised ET demonstrated significant improvement in QoL from baseline to follow-up. However, when compared to the control group at the end of the follow-up, there was significant increase in QoL in only 1[12] of those 4 studies. In another study[21] that did not have control group with LVADs, ET significantly improved QoL from baseline to follow-up (P<0.05).

**Fig 4:**
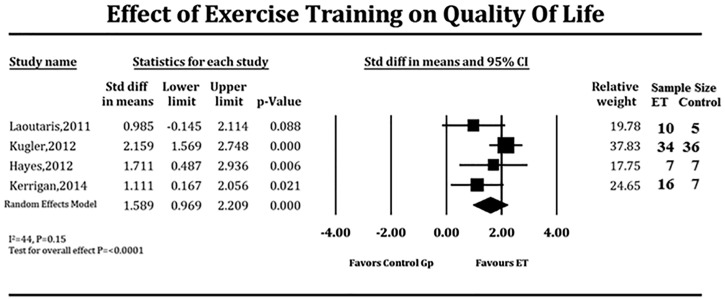
Forest plot depicting effect of supervised exercise training on quality of life in LVAD patients. ET = exercise training, Gp = group.

Due to the strong likelihood of small sample size influencing results in these individual studies, we looked at studies that specifically reported the effect of supervised ET on QoL at baseline and at follow-up in both treatment and control groups that did not undergo supervised ET. This sub-analysis could be performed in only 4 studies (Table 4). LVAD patients who underwent supervised ET demonstrated significant improvement in QoL when compared to those who did not (SMD 1.58, 95% CI 0.97–2.20 units of the standard deviation, P <0.001, Fig 4), which translated to a 12-point improvement in the supervised ET group over the control group. There is no significant heterogeneity among the trials (*I*^*2*^ = 43.32, P = 0.15). Meta-regression demonstrated no significant effect of location of supervised ET (coefficient = 0.29, P = 0.60), inclusion of strength training in the regimen (coefficient = 0.03, P = 0.86), and the dose of aerobic ET (coefficient = 0.14, P = 0.70 on the results of the pooled SMD. However, the duration of ET did show significant effect on the results of pooled SMD (coefficient = 4.21, P = 0.04).

### Adverse events and safety of ET

None of the pooled studies reported major adverse events of ET. One study[12] reported syncope in a patient immediately after completing exercise session. One study[22] reported an episode of non-sustained ventricular tachycardia during ET. However, we did not find any relation between the dose of aerobic ET and adverse events (Table 1).

## Discussion

We performed this systematic review due to recent increased referral of patients with LVADs to our cardiac rehabilitation center. There are no exercise prescription guidelines for patients with LVADs. Our systematic review suggests that supervised ET in LVAD patients is safe with no major adverse events. Supervised ET improved exercise capacity but we did not find consistent evidence to support an increase in exercise capacity or health-related QoL outcomes in LVAD patients receiving supervised ET when compared to usual care. This is likely due to small sample size and short duration of ET. However, our sub-analysis involving larger sample demonstrated that supervised ET for at least 3 days a week in LVAD patients significantly improved exercise capacity and QoL when compared to usual care. These results indicate that supervised ET has potential to improve patient outcomes in LVAD patients compared to usual care.

Our study builds on previous results since none of the individual RCTs [12, 18, 19] has shown significant improvement in exercise capacity in LVAD patients undergoing supervised ET when compared to LVAD therapy alone. Multiple exercise modalities in various doses in the treatment group in these RCTs could have influenced the peak oxyen uptake at the end of the follow-up. Low sample size in the 3 RCTs [12, 18, 19] could have contributed to the lack of statistical significance between ET and control groups, which was overcome with our sub-analysis. LVAD patients are profoundly deconditioned for prolonged periods prior to implantation. But the duration of ET in most studies was relatively short (2–12 weeks) to achieve any reasonable results. Furthermore, control groups in the 3 RCTs [12, 18, 19] exercised without any restriction and this may have influenced the difference between the treatment and control groups at follow-up. Despite the addition of a prospective non-randomized controlled study in our sub-analysis, we were able to show consistency of these findings and lack of significant heterogeneity, despite variations in exercise regimens, locations (home or rehab facility) and intensity of the ET protocols.

This study underlines the importance of ET after LVAD implantation since improvements in exercise capacity do not occur spontaneously. Indeed, Leibner and coworkers[5] demonstrated no statistical improvement in peak oxygen uptake at any time point after LVAD implantation. In their study cohort,[5] the maximal peak oxygen uptake was 12.7 ml/kg/min at 3 to 6 month period after LVAD implantation. These numbers are still lower than the criteria for severe functional limitation set for heart transplantation (peak oxygen uptake: 14 ml/kg/min).[5, 26]

There are several mechanisms to explain the beneficial effects of supervised ET. It has been shown that in addition to low cardiac output, skeletal muscle dysfunction plays a critical role in impaired exercise tolerance in HF patients.[6] Leg muscle volume correlates with exercise limitation in HF.[27] Muscle abnormalities in HF are not only caused by decreased blood flow and muscle atrophy but also increased sympathetic tone and free radical activity.[28, 29] A structured regimen of ET under supervision to ensure compliance has the potential to improve muscle strength, respiratory efficiency, peripheral vasodilation and skeletal muscle oxidative capacity.[11] The aerobic portion of ET benefits exercising limbs of patients with HF reducing sympathetic tone and lactate accumulation, improves oxidative capacity and leads to increased blood flow to the skeletal muscle, [7, 12, 30–32],[33] which will ultimately result in an increase in peak oxygen uptake, as seen in our sub-analysis. In HF patients, exercise has been shown to have minimal effect on central hemodynamics and studies demonstrated that its predominant effect is on peripheral musculature.[29, 34, 35]

The other mechanism for the improvement in study outcomes related to ET is increase in muscle strength. Muscle strength is necessary to perform short-duration activities of daily living (like dressing and showering), which enhances QoL.[7] LVAD patients often have difficulty in returning to their routine activities, which are known predictors of poor QoL.[18] The incorporation of resistance training as part of the regimen can greatly improve QoL. This was seen in one of the pooled studies,[18] which included upper and lower extremity strength training exercises. Even when resistance training was not a part of the regimen, leg muscle strength still improved with structured ET as shown in one[12] of the studies in this systematic review. It is likely due to the fact that the supervision of ET resulted in a greater workload than just usual care.

It is also important to emphasize the outcome effects of the duration of ET despite a non-significant trend in the relationship between exercise duration and the improvement in exercise capacity (P = 0.07) over usual care. In fact, the study with the longest training duration (Kugler et al, 18 weeks)[13] was also the one that showed highest improvement in peak oxygen uptake and QoL when compared to usual care. Our meta-regression analysis supports this hypothesis by demonstrating significant association of duration of ET with QoL.

There are several limitations in this systematic review. Our analysis includes only 8 studies reflecting the severe scarcity of data on our chosen topic possibly due to challenges in recruiting, and difficulty in performing supervised ET in LVAD patients. The follow-up of 3 RCTs [12, 18, 19] is relatively short and therefore we could not capture long term hard outcomes like hospitalization or death. Despite each of the pooled studies employing supervised exercise protocols, the variation in the type, intensity, and the location of the ET protocols may have influenced the precision of our results. Furthermore, 4 studies did not report INTERMACS levels which is a useful tool in stratifying patient’s illness severity before LVAD implantation. This missing INTERMACS data may have limited our ability to quantify the heterogeneity of the patient’s characteristics in the interpretation of the result, albeit not statistically significant (*I*^*2*^ = 11.48, P = 0.33). Supervised ET was initiated at different time points after the LVAD implantation in the pooled studies and this time-dependent variable may have influenced the peak oxygen uptake values, although our meta regression analysis ruled out this influence. Our systematic review includes patients that underwent ET soon after the LVAD implantation (<10 weeks) and during this time period, patients are still severely deconditioned and this factor may have influenced the benefits of ET. Therefore, our estimates of the peak oxygen uptake may have underestimated the maximal benefits of exercise training. We anticipate criticism that multifaceted intervention strategies like phone calls, medication optimization, psycho-social support, behavioral modification, and feedback could have biased the true effect of ET in the treatment group but one of the main purposes of this systematic review is to find the gaps in the current literature for future research. We acknowledge the potential risk of confounding and selection bias due to the inclusion of a non-randomized prospective study [13] in the sub-analysis despite robust results of our sensitivity analysis and minimal statistical heterogeneity. The studies used different scales to measure QoL which could have impacted the effect size despite our use of SMD methodology that overcomes the measurement barrier in theory. Exercise testing using different modalities (treadmill versus bicycle ergometer) could have influenced the peak oxygen uptake values.[20] Multiple studies [36–40] have shown that the peak oxygen uptake of leg cycling is approximately 89% to 95% of the maximal values achieved with treadmill exercise, which should be taken into account in the interpretation of the results. Adjustment of the LVAD speed in the continuous-flow devices in one study [13] could have influenced the peak oxygen uptake. The inclusion of pulsatile-flow LVADs in two[19, 20] of the pooled studies may have influenced our results, however, Haft and coworkers [41] demonstrated that that exercise performance at 3 months after LVAD implantation with a pulsatile flow device was not significantly different from exercise performance achieved by continuous flow devices. This study showed that no significant differences in peak oxygen uptake were observed despite significant differences in left ventricular volume unloading between different LVAD designs suggesting that major differences in LVAD design do not significantly influence exercise performance. The inclusion of destination therapy patients in one study [23] could have biased the results of this review as destination therapy patients are much sicker with more comorbidities and worse survival rates.[42]

## Conclusion

Our systematic review is insufficient to provide evidence on any specific exercise training intervention or regimen in LVAD patients due to lack of consistency in study designs, sample size, and duration of ET. Our analysis of studies with a control group suggests that supervised ET has the potential to improve exercise capacity and QoL as LVAD patients remain severely deconditioned post-implantation. Duration of ET seems to be an important factor influencing QoL in LVAD patients. Therefore, long-term outcomes of supervised ET for LVAD patients need further investigation with larger, ideally multicenter RCTs incorporating consistent exercise protocols and a longer follow-up period.

## Supporting information

S1 File PRISMA 2009 checklist document. (DOC)

## Abbreviations

ET: Exercise training
HF: Heart Failure
ICM: ischemic cardiomyopathy
LVAD: Left ventricular assist devices
NICM: non-ischemic cardiomyopathy
QoL: Quality of life
